# Flow Injection Chemiluminescent Immunoassay for Carcinoembryonic Antigen Using Boronic Immunoaffinity Column

**DOI:** 10.3390/s91210389

**Published:** 2009-12-21

**Authors:** Xiaoling Ma, Huihui Li, Min Wu, Chong Sun, Laifa Li, Xiaodi Yang

**Affiliations:** 1 Nanjing Maternity and Child Health Care Hospital, Nanjing Medical University, Nanjing, 210004, China; E-Mail: xiaolingma2003@yahoo.com.cn; 2 Jiangsu Key Laboratory of Biofunctional Materials, College of Chemistry and Environmental Science, Nanjing Normal University, Nanjing, 210097, China; E-Mails: huihuili@njnu.edu.cn (H.H.L.); wumin@njnu.edu.cn (M.W.); sunchong0106@163.com (C.S.); lilaifa@njnu.edu.cn (L.F.L.)

**Keywords:** flow injection chemiluminescence immunoassay, carcinoembryonic antigen, boronic immunoaffinity column

## Abstract

A flow injection chemiluminescence immunoassay for rapid and sensitive detection of carcinoembryonic antigen (CEA) by using a phenylboronic acid-based immunoaffinity column as a glycoprotein collector was proposed in this paper. The column was prepared by coupling of 3-aminophenylboronic acid on the glass beads through a γ-glycidoxypropyltrimethoxysilane (GPMS) linkage. Based on an indirect competitive immunoreaction, the mixture of CEA sample and enzyme conjugated CEA antibody (HRP-anti-CEA) was incubated in advance, followed by direct injection to the column to capture free HRP-labeled CEA antibody in the column. The trapped HRP-labeled antibody was detected by flow inject chemiluminescence in the presence of luminol and hydrogen peroxide. The decreased chemiluminescent signal was proportional to the concentration of CEA in the range of 3.0–30.0 ng/mL with a correlation coefficient of 0.998. The column showed an acceptable reproducibility and stability and is potentially used for practical clinical detection of the serum CEA level.

## 1. Introduction

Carcinoembryonic antigen (CEA) is a highly glycosylated cell surface glycoprotein with a total of 28 asparagine-linked glycosylation sites [1]. It has been used as an important tumor marker in disease diagnosis for colorectal, pancreatic, gastric, and cervical carcinomas [2]. Presently, enzyme-linked immunoassays [3-4], fluorescence measurement [5-7] and chemiluminescence assay [8] are frequently used to detect to successfully detect the CEA in practical samples with high selectivity, sufficient sensitivity and precision. However, these conventional immunoassay methods need enzyme or fluorescent-labeled antibody/antigen, not to mention its lengthy analysis that requires highly skilled personnel, specially equipped laboratories, and expensive chemicals [9-11]. Therefore, it is highly desirable for disease diagnosis to develop new methods for fast and convenient monitoring of CEA.

Herein we adopted a boronic immunoaffinity column as glycoprotein antibody collector in combination with flow injection immunoassay to develop a novel detection method for CEA. Alkyl or aryl boronic acids can react with vicinal diols to form boronate complexes [12]. The boronate groups could be immobilized on solid surface or in gel for recognition and separation of glycoproteins [13]. In this work, 3-aminophenylboronic acid was coupled on the glass microbead surface for construction of a boronic immunoaffinity column. The immobilized APBA on glass microbeads was prepared for accumulation of glycoprotein antigen CEA, followed by the recognition of free horseradish peroxidase (HRP)-labeled CEA antibody in the mixture of the analyte CEA and HRP-labeled CEA antibody after incubation. The trapped HRP-labeled CEA antibody enhanced the chemiluminescence emission intensity of luminal system, which is the basic for detection of the analyte CEA in samples.

## 2. Results and Discussion

### 2.1. Binding performances of the immunoaffinity column

**Figure 1.**
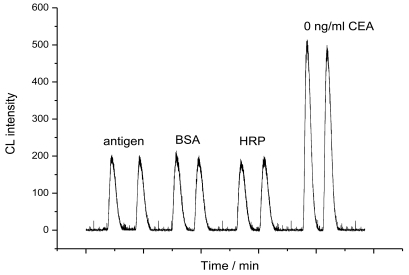
Control experiments by flow of 100 μL of 0 ng/mL CEA without enzyme-labeled, 0.1 mg/mL BSA, 2 mg/mL HRP and 9:1 of HRP-labeled anti-CEA and CEA standard solution containing 0 ng/mL after being incubated at room temperature for 25 min. The substrate solution is 0.5 mM luminol + 4 mM H_2_O_2_ + 0.4 mM PIP.

**Scheme 1.**
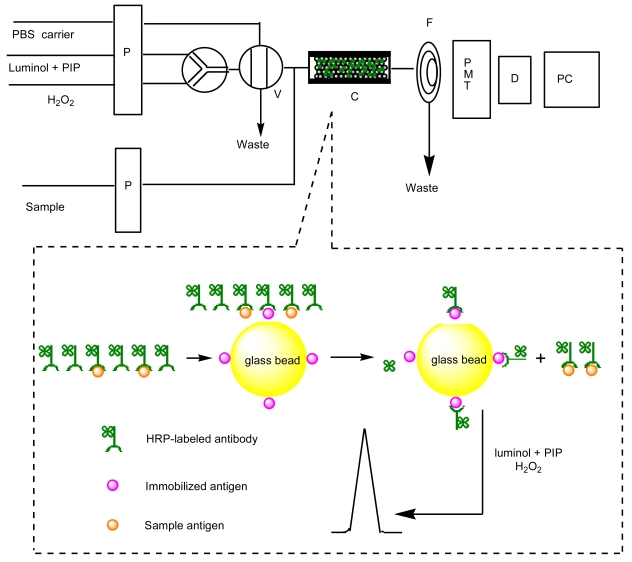
Schematic diagram of flow injection immunoassay system: (P) peristaltic pump, (V) eight-way valve, (C) immunoaffinity column, (PMT) photo-multiplier, (D) detector, (PC) computer.

**Scheme 2.**
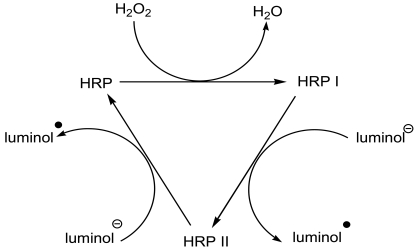
The mechanism for the CL emission enhancement through HRP.

The 3-aminophenylboronic acid (APBA) coated glass microbeads were prepared by using γ-glycidoxypropyltrimethoxysilane (GPMS) as linkage. Then the as-prepared APBA modified microbeads were filled into a glassy tube followed by injection of a 250 ng/mL CEA solution into the column to form the immunoaffinity column. After that the column was connected to a spiral glass tube for the chemiluminescence measurements. The preparation of the immunoaffinity column and the detection process were shown in Scheme 1. The binding performances of CEA to the APBA modified glass microbeads were characterized by chemiluminescence and photometric activity measurements. Previous studies show that the chemiluminescence intensity of the luminal + H_2_O_2_ system can be enhanced by HRP-labeled antibody [14]. It is the basis of the immunosensors designed here that HRP-labeled antibodies is used as a CEA tracer for quantification of proteins. The mechanism for this emission enhancement through HRP was illustrated in Scheme 2. After being incubated in 90 μL HRP-anti-CEA and 10 μL CEA at 37 °C for 25 min, the mixture was injected to the column, which has been washed with PBS to establish the baseline before. Upon injection of luminal + *p*-iodophenol (PIP) + H_2_O_2_ to the column, the chemiluminescence emission intensity detected behind the column (5 cm) was about 2.5 times higher than that of the column not exposed to HRP-labeled antibodies (Figure 1). The enhanced emission indicated that there was the successful trapping of HRP-labeled antibodies by the immobilized antigen, which sensitized the reaction of luminol and hydrogen peroxide.

In the control experiments using bovine serum albumin (BSA), HRP, instead of HRP-labeled CEA antibody to inject into the bioreactor, no enhanced CL intensity can be observed (Figure 1). The HRP is known as a glycoprotein with degree of glycosylation of 16–21% [15]. Therefore, it is possible that there are two binding modes reacting with the APBA interface directly and/or replace CEA on the APBA-modified surface, consequently resulting in a nonspecific increase of the chemiluminescence emission intensity. Indeed, when injection of free HRP into the column filled with APBA-modified glass beads without CEA, an increase of the chemiluminescence emission intensity was observed (data not shown). After blocking the APBA-modified glass beads with CEA, however, injection of free HRP did not exhibited clearly enhancement of the chemiluminescent emission intensity (Figure 1). This result showed a much weak-binding interaction between HRP and APBA after saturating the APBA interface with CEA. Thus, the increase of the emission intensity in the previous experiment is attributed to the presence of HRP-labeled antibody on the CEA-modified surface, which quantitatively correlates to the amount of free CEA in the sample.

The specific formation of immunocomplex of HRP-labeled CEA antibody and CEA was further demonstrated by photometric activity measurements using tetramethyl benzidine (TMB) as substrate [12]. After reacting with HRP labeled anti-CEA, the glass beads were added to TMB solution containing H_2_O_2_, there is a large absorbance observed at 654 nm. Adding the CEA modified glass beads to the above detection solution without incubation, no clear absorbance at 654 nm was observed. This illustrated that the immobilized CEA on the glass beads could bind with HRP-labeled anti-CEA thorough immunoreaction and blocked free HRP close to the APBA interface.

### 2.2. Non-competitive immunoassays for CEA detection

**Figure 2.**
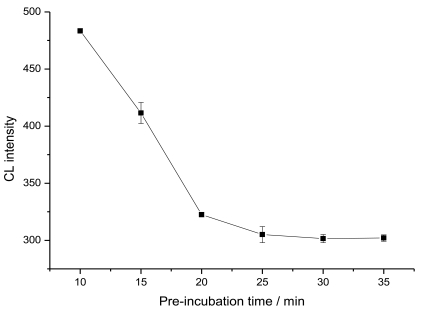
Effect of the pre-incubation time on CL intensity of 0 ng/mL CEA 1:9 mixed with enzyme tracer.

The determination of CEA was based on the non-competitive immunoassay. A phosphate buffer solution (PBS, 0.1 M, pH 7.0) was used as the carrier. Ninety μL of HRP-labeled anti-CEA and 10 μL of CEA standard solution were mixed and loaded on the immunoaffinity column at a flow rate of 0.05 mL/min, followed washing with PBST (pH 7.0, containing 0.05% Triton X-100) to remove the unreacted HRP-labeled anti-AFP and physically adsorbed enzyme. Then luminol, PIP and H_2_O_2_ were mixed and delivered to the column with the aid of the valve; the CL signal was detected. To optimize the pre-incubation time, 10 μL 0 ng/mL CEA was incubated with 90 μL HRP-labeled antibody at 37 °C, detected at different time intervals, as shown in Figure 2. At the pre-incubation time of 25 min, the CL intensity decreased to a minimum value, indicating a maximum combination of CEA with its enzyme tracer. Thus, a pre-incubation of 25 min was chosen for the present system.

**Figure 3.**
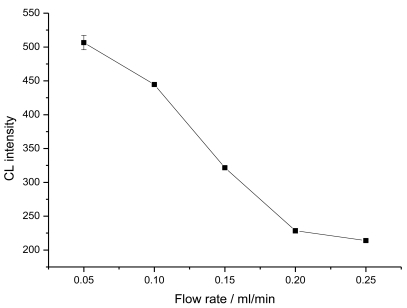
Effect of flow rate of pre-incubation immunomixture through the immunoaffinity column on the CL intensity. Pre-incubation time of 0 ng/mL CEA with enzyme tracer was 25 min

Figure 3 shows the effect of the flow rate on the CL intensity. The relative low flow rate is in favor of trapping free enzyme tracer in column. The result showed that the lowest flow rate from our pump was 0.05 mL/min, which was selected as the flow rate of the immunomixture.

**Figure 4.**
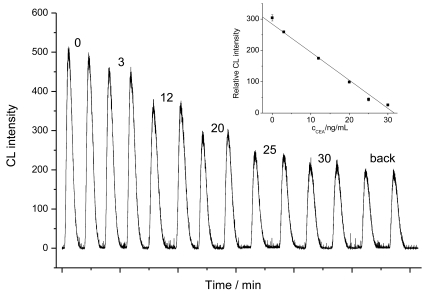
Dose-response curve for CEA. Inset: Plots of CL intensity versus CEA concentration in pre-incubation solution.

The CL intensity was decreased with the increasing CEA concentration as shown in Figure 4, showing a linear range of 3–30 ng/mL with a correlation coeffience of 0.998 (insert in Figure 4). The regression equation was *I* = 284.38 – 8.97*c* (ng/mL), where *I* is the relative CL intensity and *c* is the CEA concentration. When the CEA concentration was increased up to 30 ng/mL, an appropriate dilution of sample was needed in the pre-incubation step.

**Figure 5.**
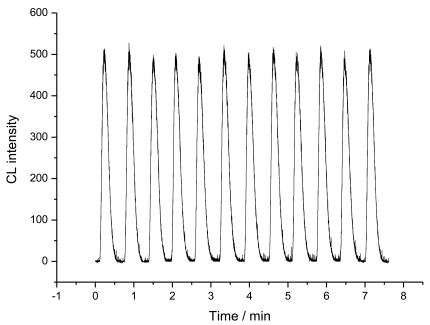
The CL intensity of 0.5 mM luminol + 4 mM H_2_O_2_ + 0.4 mM PIP at CEA concentration of 0 ng/mL after regeneration of the immunoaffinity reactor by 10 mM NaOH.

**Figure 6.**
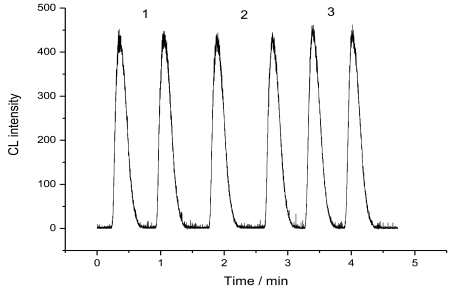
Reproducibility of the immunoaffinity reactor, 0.5 mM luminol + 4 mM H_2_O_2_ + 0.4 mM PIP at CEA concentration of 3 ng/mL.

Regeneration properties of the column are very important to a flow-injection immunoassay system. Due to the low binding stability of glycoprotein and boronic acid in acidic solutions, the alkaline solution of 10 mM NaOH, was used in the present system to disrupt the antigen-antibody complex. The column was flowed by 10 mM NaOH solutions for 1 min, and then equilibrated with 0.1 M PBS (pH 7.0) for 1 min. The chemiluminescence emission intensity was decreased to its back value due to the release of the bound HRP-labeled CEA antibody. After reinjection of enzyme conjugated CEA antibody to the column followed by injection of luminal + PIP + H_2_O_2_ to the reactor, the chemiluminescence emission intensity was increased to the value before regeneration, indicating the rebinding of enzyme conjugated CEA antibody with CEA on the support surface. At one immunoaffinity column, the mean steady-state CL intensity was 510 with a relative standard deviation of 1.95% for twelve determinations at 0 ng/mL of CEA in the pre-incubation solution with 0.5 mM luminol + 4 mM H_2_O_2_ + 0.4 mM PIP as substrates as shown in Figure 5. When the immunoassay column was not in use, it was stored in PBS (pH 7.0) at 4 °C. No obvious change was found after 15 days. The fabrication reproducibility of three column, made independently, exhibited an acceptable reproducibility with a relative standard deviation of 1.81% for the CL intensity determined at 3 ng/mL of CEA in the pre-incubation solution with 0.5 mM luminol + 4 mM H_2_O_2_ + 0.4 mM PIP as substrates (Figure 6). This indicated that the immunoassay column possess good reproducibility and could be used repeatedly. The whole assay process including regeneration of the reactor could be achieved in 31 min. The total analytical time was shorter than that of 40 min with amperometric immunosensor [16], more than 1 h with the conventional immunoassay methods, including radioimmunoassay, single radial immunodiffusion, immuno-turbidimetry and enzyme-linked immunoassay [17,18].

The serum CEA levels in five samples were detected using the proposed flow injection chemiluminescence immunoassay. The CEA concentrations in the clinical serum of some patients were beyond the linear range of the described method; thus proper dilution with 0.85% NaCl before assay was necessary. The average concentrations of the serum CEA samples were determined to be 31.9, 83.3, 3.7, 5.9 and 223.6 ng mL^-1^, respectively. The results are compared with those of 29.6, 81.9, 3.8, 5.2 and 200.5 ng mL^-1^ obtained using a standard method provided by Jiangsu Institute of Cancer Prevention and Cure, respectively. The relative deviations are in the range from 7.2 to 12.1% between the two methods, which was considered as acceptable.

## 3. Materials and Methods

Carcinoembryonic antigen and horseradish peroxidase (HRP)-labeled CEA antibody (HRP-anti-CEA) were purchased from CanAg Diagnostics AB. Bovine serum albumin (BSA), horseradish peroxidase, 3-aminophenylboronic acid were obtained from Sigma-aldrich Chemical Company (Shanghai, China). γ-Glycidoxypropyltrimethoxysilane was provided by Jintan Huadong Coupling Agent Co., Ltd. (Jiangsu, China). Glass microbeads (80-mesh) were purchased from Shanghai Chemical Plant (Shanghai, China). *p*-Iodophenol was purchased from Weihai Newera Chemical Co. Ltd. (Shandong, China). All other chemicals were of analytical grade and used without further purification. Deionized water was used throughout the study. A stock solution of 0.01 M luminol was prepared by dissolving 177 mg of luminol in 100 mL of 1 M NaOH and was stored in dark. The stock solution of 0.01 M PIP was made by dissolving 220 mg PIP in 10 mL of dimethylsulfoxide (DMSO) and then diluted with water to 100 mL, followed by storage in dark. Prior to the use, both luminol and PIP solutions were diluted with 0.1 M Tris-HCl buffer solution (pH 8.5) to the desired concentrations.

Five grams of glass microbeads were boiled in 5% HNO_3_ for 1 h. After washing thoroughly with water and drying at 130 °C for 4 h, the activated glass beads were reacted with 100 mL of 5% GPMS in dry toluene at room temperature under stirring overnight. Then they were washed with toluene and ethanol and dried under a nitrogen atmosphere at 100 °C for 1 h. After that the beads were soaked in 6 mL 10 mg/mL APBA solution under stirring at 4 °C for 24 h. The column was fabricated by filling the APBA modified microbeads into a glassy tube (1.6 mm inner diameter and 5 cm length). For CEA immobilization, 300 μL 250 ng/mL CEA solution was injected to the column and incubated for 1 h at 4 °C. The CEA immobilized column was then connected to a spiral glass tube (2.0 mm inner diameter and 10 cm length) for the follow experiments.

The determination of CEA was based on a non-competitive immunoassay. A phosphate buffer solution (PBS, 0.1 M, pH 7.0) was used as the carrier. 90 μL HRP-labeled anti-CEA and 10 μL CEA standard solution were mixed and incubated at 37 °C for 25 min. After a stable baseline was recorded, the mixture was loaded on the immunoaffinity column at a flow rate of 0.05 mL/min. The column was washed with PBST (pH 7.0, containing 0.05% Triton X-100) for 1 min at a rate of 1.0 mL/min to remove the unreacted HRP-labeled anti-AFP and physically adsorbed enzyme. Then luminol, PIP and H_2_O_2_ were mixed and delivered to the column with the aid of the valve; the CL signal was detected with IFFM-E Luminescence Analyzer (Remax, China). The modified glass beads were regenerated with a flow of 10 mM NaOH for 1 min, and then equilibrated with 0.1 M pH 7.0 PBST for 1 min. The total assay time was 31 min, including 25 min of pre-incubation and 6 min of the detection and regeneration.

## 4. Conclusions

In summary, we developed a rapid and sensitive immunoassay based on a phenylboronic acid immunoaffinity column in combination with a flow injection chemiluminescence (CL) for determination of glycated antigen protein. With the sugar-boronic interaction, the CEA could be easily immobilized on APBA coated glass beads. After an off-line incubation, the free HRP-labeled antibody in the mixture of the analyte CEA with HRP-labeled AFP antibody was captured by the immobilized antigen in the column. The trapped HRP-labeled antibody on column enhanced the chemiluminescence emission intensity of the reaction of luminol and hydrogen peroxide, which quantitatively correlates to the amount of free CEA in the sample. The whole assay process including regeneration of the reactor could be achieved in 31 min. The described method was shown as a acceptable detection with fabrication reproducibility. It is proven to be fast and simple and could be further developed for practical clinical detection of serum CEA levels.
